# Diabetes Mellitus as a Risk Factor for Trigger Finger –a Longitudinal Cohort Study Over More Than 20 Years

**DOI:** 10.3389/fcdhc.2021.708721

**Published:** 2021-11-02

**Authors:** Jin Persson Löfgren, Malin Zimmerman, Lars B. Dahlin, Peter M. Nilsson, Mattias Rydberg

**Affiliations:** ^1^ Department of Translational Medicine, Hand Surgery, Lund University, Malmö, Sweden; ^2^ Department of Hand Surgery, Skåne University Hospital, Malmö, Sweden; ^3^ Department of Clinical Sciences, Lund University, Malmö, Sweden; ^4^ Department of Emergency and Internal Medicine, Skåne University Hospital, Malmö, Sweden

**Keywords:** trigger finger, stenosing tenosynovitis, diabetes mellitus, registries, diabetic hand

## Abstract

**Background and Aim:**

Trigger finger (TF) or stenosing tenosynovitis has been associated with diabetes mellitus (DM), although today’s knowledge is mostly based on cross-sectional and case-control studies. Thus, the aim of the present population-based cohort study over more than 20 years was to investigate DM as a risk factor for TF.

**Methods:**

Data from Malmö Diet and Cancer Study (MDCS), including 30,446 individuals, were analysed with regards to baseline DM and known or potential confounders. Information regarding TF diagnosis until study end date of Dec 31^st^, 2018, was retrieved from the Swedish National Patient Register (NPR) using ICD-codes. Survival probability was investigated in Kaplan-Meier plots. Cox proportional hazard regression model was used to evaluate DM as risk factor for TF, adjusted for several confounders and presented as Hazard Ratio (HR) with 95% confidence intervals (CI).

**Results:**

At baseline, 4.6% (1,393/30,357) participants had DM. In total, 3.2% (974/30,357) participants were diagnosed with TF during the study period. Kaplan-Meier plot showed that the probability for incident TF was significantly higher in participants with baseline DM compared with individuals without baseline DM. Adjusted HR for DM as risk factor for TF was 2.0 (95% CI: 1.5-2.6, p<0.001).

**Conclusion:**

This longitudinal study showed that DM is an important risk factor for developing TF. When adjusting for sex, age, BMI, manual work, statin use, smoking and alcohol consumption, DM remained the main risk factor for TF.

## Introduction

Diabetes Mellitus (DM) is one of modern time’s most challenging long-term public health challenges, both in terms of individual suffering and health care economics. The number of patients diagnosed with diabetes mellitus type 1 (T1D) and type 2 (T2D) is rapidly increasing (1). Complications caused by DM include a wide variety of disorders, where cardiovascular complications, nephropathy, neuropathy, and retinopathy are the most studied. Less studied is *“the diabetic hand”*, which includes trigger finger (TF), Dupuytren’s disease with contracture of the finger joints (DC), limited joint mobility (LJM), carpal tunnel syndrome (CTS) and ulnar nerve entrapment (UNE) (2). Individuals with DM are also more likely to suffer from bilateral involvement and multiple disorders (3).

Trigger finger (TF), also known as stenosing tenosynovitis, tenovaginitis or digitus saltans, is a condition where the flexor tendon is obstructed in its tendon sheath at the first annular (A1) pulley. This results in a locking phenomenon, and the affected finger can only be extended with additional force or passive manipulation, which can be painful. The thumb and the ring finger are most affected, followed by the long finger (4–6). There is no known cause of TF, and its pathogenesis is not completely defined (7). Treatment options include intra- or extra synovial corticosteroid injection and percutaneous or open surgical release of the A1 pulley (7–9).

The prevalence of TF is approximately 1-2% in the general population (10, 11). Women are affected twice as often as men and prevalence peaks in ages 50 to 59 years (10). Prevalence rates and the risk for TF in individuals with DM are not conclusive, ranging from 1.5% to 20% depending on the group studied (5, 11–13). Our aim of the present population-based cohort study over more than 20 years, was to investigate the impact of DM as a risk factor for TF, using the large Malmö Diet and Cancer study (MDCS) cohort in southern Sweden.

## Materials and Methods

### Study Design

In the Malmö Diet and Cancer Study (MDCS) cohort, participants with DM at baseline were identified. Incident TF diagnosis during the study period was identified using the Swedish National Patient Register (NPR). The outcome, using the statistical method described below, was the probability for incident TF in participants with baseline DM compared with individuals without baseline DM. In this study, we did not use data collected during and/or at the end of the study period, therefore exposure during the study period was not included. Potential confounders included in this study are age, sex, BMI, manual work, statin use, smoking habits, and alcohol consumption. Potential sources of bias include selection, detection and reporting bias and are described in more detail in section 4.1 Strengths and Limitations.

### Study Population

The present data was retrieved from the Malmö Diet and Cost Study (MDCS) cohort. The initial objective with MDCS was to study the association between diet and development of cancer (14). Participants in the MDCS were recruited during 1991-1996 in Malmö, a city in southern Sweden of approximately 250 000 inhabitants (15). Participants were 44-74 years old when recruited; 60% being women. Participants provided information regarding their work life, socio-economic situation, heredity, lifestyle, diet, and medical history in a questionnaire, a 7-day food diary, and a 45-60 min diet history interview. Blood samples as well as blood pressure, height and weight, lean body mass and body fat mass, were collected at baseline.

### Baseline Definitions

Age was defined as the participant’s age at enrollment in the MDCS. Body mass index (BMI) was calculated from data collected at enrollment and expressed in kg/m^2^. Prevalent DM was defined based on the participant’s questionnaire, if medical history stated a DM diagnosis or the use of antidiabetic medication, or if the participant had fasting plasma glucose concentration ≥ 7.0 mmol/L. Information regarding baseline DM was also retrieved from several other registries, previously described in detail (16). Manual work was based on free text answer in the participant’s questionnaire and classified using the Nordic standard occupational classification (17), which has been previously described for the MDCS cohort (15). Statin use included simvastatin, pravastatin and fluvastatin and was based on the participant’s questionnaire and the 7-day food diary. Smoking habits were collected from the participant’s questionnaire. Current smokers were defined as regular or occasional smokers. Previous smokers were split into regular and occasional smokers using the ratio from the current smoker group above, and number of cigarettes per day was assumed using the mean value of regular and occasional smokers. Smoking habits is presented as pack years; number of cigarettes smoked per day divided by 20 multiplied by numbers of years smoked. Alcohol consumption was based on the participant’s questionnaire and was converted into g/day.

### End Point Definition

End point was either incident TF diagnosis, death, emigration, or end of study Dec 31^st^, 2018. Information regarding prevalent and incident TF diagnosis was retrieved from the Swedish National Patient Register (NPR). International Statistical Classification of Diseases and Related Health Problems (ICD) version 8, 9 or 10 codes 731.02, 727X, 727A and M653 were used as diagnosis codes for TF. Diagnosis was made by hospital-based physicians, mainly by orthopaedic and hand surgeons, whereas TF diagnoses from primary health care were not included in NPR.

### Statistical Methods

Participants with a prevalent TF diagnosis at baseline or with missing information regarding start date and/or BMI were excluded from further analysis. Age was normally distributed and presented as mean with standard deviations (SD). When comparing age in the group free from incident TF with the group with incident TF, the independent t-test was used. BMI, pack years for smokers and previous smokers, and alcohol consumption were not normally distributed and are presented as median with interquartile range [IQR]. For these parameters, the Mann-Whitney U test was used when comparing the incident free group with the incident TF group. For categorical variables, i.e., sex, prevalent DM, manual work and statin use, proportion (%) was used, and the Chi-squared test was used for group comparisons.

Data was analysed and presented as survival probability and hazard probability, using the Kaplan-Meier and Cox proportional hazard (PH) regression methods (18). A log-rank test was used to compare survival probability for participants with prevalent baseline DM with those without prevalent baseline DM. However, the Kaplan-Meier method gives no estimate of the actual impact of DM, and there is no possibility to assess the impact of confounders. Thus, Cox proportional hazard (PH) regression model was also used in the survival analysis (19), where hazard ratio (HR) was reported with a 95% confidence interval (CI). The included confounders were sex, age, BMI, manual work, statin use, alcohol consumption and smoking habits. Sex and age were selected to adjust for differences in the compared groups. BMI is a potential risk factor for TF (20, 21) as well as statin use (22, 23). HR was firstly assessed for each covariate in separate univariate Cox PH regression models. Then, several multivariate Cox PH regression models were used to investigate how the covariates would affect the HR for incident TF in relation to prevalent baseline DM. The assumption of proportional hazard was assessed by log-log plots and visual assessment of Kaplan-Meier curves and no violation was found.

All statistical analyses were performed using IBM SPSS Statistics version 26 (SPSS Inc., Chicago, IL, USA) and p < 0.05 was considered significant.

### Ethics Approval Statement

For both this study and the original study, the ethical application was approved by the Regional Ethical Review Board in Lund, Sweden (DNR: LU51-90; 2009-633; 2019-01439) and carried out in accordance with the World Medical Association’s Declaration of Helsinki.

## Results

**Figure 1:**
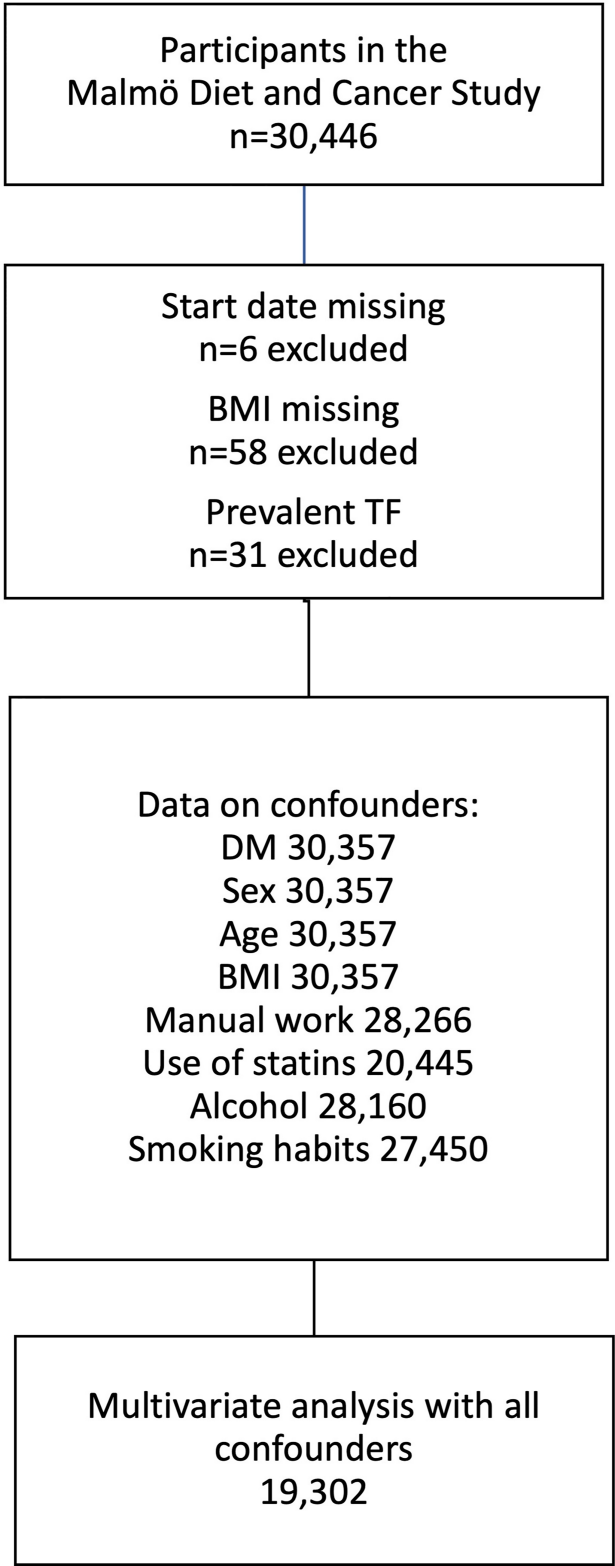
Derivation of the study cohort from Malmö Diet and Cancer study. Flow chart showing exclusion criteria and data availability for individuals included in the multivariate analysis. TF, Trigger finger; BMI, body mass index; DM, diabetes mellitus.

The total number of participants in the MDCS was 30,446. Participants, where start date and/or BMI were missing, were not included in further analysis (*n*=58). The same was applied for participants who were already diagnosed with TF (*n*=31) when they were recruited to the MDCS (**Figure 1**).

### Baseline Characteristics

Mean age for all individuals was 57.5 (SD 7.6) years and 40% (12,085/30,357) were male. BMI was median 25.8 [5.0] kg/m2. At baseline, there were 1,393/30,357 (4.6%) participants with prevalent DM.

In the MDCS, information regarding statin use was available for 67% (20,445/30,357) and 3.2% (655/20,445) used statins. Information regarding manual work was available for 93% (20,266/30,357) of the participants and 38% (10,631/20,266) were classified as manual workers, Data regarding smoking habits defined as pack years was available for 90% (27,450/30,357) of the cohort, and smoking participants had smoked for a median of 6.8 [18.4] pack years. Data on alcohol consumption was available in 93% (28,160/30,357), and median alcohol consumption was 7.2 [13.7] g/day.

### End-Point Data

In total, 263 individuals left the study before end date of Dec 31^st^, 2018, due to emigration, and 12,609 individuals passed away during the study period. Median follow-up time for individuals without prevalent DM at baseline was 23.3 [7.1] years. For individuals with prevalent DM at baseline, median follow-up time was 18.3 [12.6] years.

### Results for Trigger Finger

In total, 974 individuals were diagnosed with TF during the study period. Individuals with incident TF were younger, more likely to be female, had higher BMI and more often had DM. There were no differences in proportion with manual work, statin use, alcohol consumption and smoking habits between the two groups (**Table 1**).

**Table 1:** Characteristics for individuals in the Malmö Diet and Cancer Study, without trigger finger (TF) and with incident TF.

Characteristics	All individuals (n = 30,357)	Without TF (n = 29,383)	Incident TF (n = 974)	P-value*
Age, years (SD)	57.5 (7.6)	57.6 (7.6)	55.6 (7.4)	**<0.001**
Male sex (%)	12,085 (40)	11,788 (40)	297 (31)	**<0.001**
BMI, kg/m^2^ [IQR]	25.8 [5.0]	25.8 [5.0]	26.2 [5.0]	**0.001**
Prevalent DM (%)	1,393 (4.6)	1,317 (4.5)	76 (7.8)	**<0.001**
	(n=28,266)	(n=27,339)	(n=927)	
Manual work (%)	10,631 (38)	10,294 (38)	337 (36)	0.42
	(n=20,445)	(n=19,723)	(n=722)	
Statin use (%)	655 (3.2)	628 (3.2)	27 (3.7)	0.41
	(n=28,160)	(n=27,247)	(n=913)	
Alcohol consumption, g/day [IQR]	10.7 [13.7]	10.8 [13.8]	10.3 [12.8]	0.85
	(n=27,450)	(n=26,543)	(n=907)	
Smoking habits, pack years	11.0 [18.38]	11.0 [18.4]	10.3 [18.0]	0.59

Table showing baseline data for participants in the MDCS, comparing individuals without and with incident TF diagnosis during the study period.

*Group comparison to investigate statistical significance p < 0.05. For age, independent t-test was used. For sex, prevalent diabetes, manual work and statin use, Chi-Square test was used. For BMI, Mann-Whitney was used.

MDCS, Malmö Diet and Cancer Study; TF, Trigger finger; DM, diabetes mellitus; BMI, body mass index; SD, standard deviation; IQR, interquartile range.

Bold value means statistically significant.

**Figure 2:**
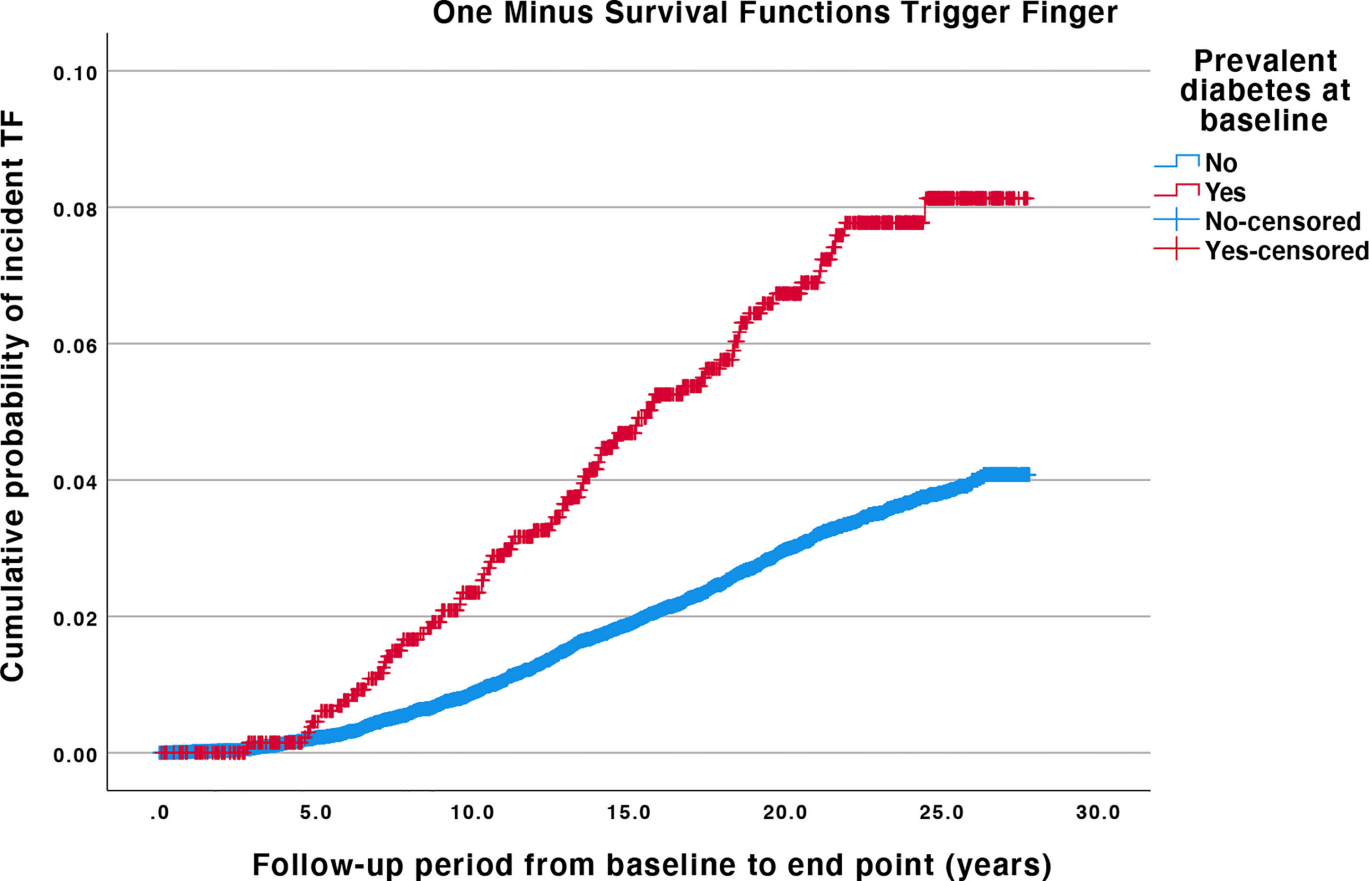
Kaplan-Meier plots for trigger finger, with and without diabetes mellitus at baseline. Log-rank test for difference in survival probability for TF with or without baseline DM is p < 0.001. The difference between the curves is proportional at all times. Individuals are censored if they leave the cohort before the end date due to emigration or death. TF, Trigger finger; DM, diabetes mellitus.

Kaplan-Meier survival probability plots showed that the cumulative probability of TF incidence was higher for individuals with baseline DM compared with individuals without baseline DM (log-rank test p < 0.0001) (**Figure 2**).

Baseline DM resulted in increased HR in the univariate Cox PH regression model (HR 2.27; 95% CI: 1.8-2.87; p<0.001). Female sex and higher BMI also resulted in an increased HR (HR 1.36; 95% CI; 1.19-1.56; p<0.001 and HR 1.03; 95% CI 1.02-1.05; p<0.001). Higher age resulted in a decrease in HR (HR 0.98; 95% CI: 0.98-0.99; p<0.001). Manual work, statin use, smoking habits and alcohol consumption did not affect HR. In the first multivariate Cox PH regression model, with DM as the covariate, HR remained significantly increased when separately adjusting for sex, age, manual work, BMI, statin use, alcohol consumption and smoking habits (**Table 2**). When simultaneously adjusting for all confounders, HR remained significantly (HR 2.00; 95% CI: 1.51-2.63; p<0.001).

**Table 2:** Cox proportional hazard regression model for trigger finger, without and with confounders.

Univariate analysis without confounders				
Covariate	Confounder	HR	95% CI	P-value
DM	–	2.271	1.797-2.871	**<0.001**
Sex	–	1.359	1.185-1.558	**<0.001**
Age	–	0.984	0.975-0.992	**<0.001**
BMI	–	1.032	1.016-1.047	**<0.001**
Manual work	–	1.013	0.886-1.158	0.855
Statin use	–	1.399	0.953-2.056	0.087
Alcohol consumption	–	0.996	0.991-1.002	0.215
Smoking habits	–	1.002	0.997-1.007	0.348
**Multivariate analysis with one confounder**				
**Covariate**	**Confounder**	**HR**	**95% CI**	**P-value**
DM	Sex	2.363	1.869-2.989	**<0.001**
DM	Age	2.387	1.886-3.021	**<0.001**
DM	BMI	2.128	1.677-2.699	**<0.001**
DM	Manual work	2.283	1.791-2.909	**<0.001**
DM	Statin use	2.130	1.643-2.761	**<0.001**
DM	Alcohol consumption	2.219	1.733-2.842	**<0.001**
DM	Smoking habits	2.249	1.759-2.875	**<0.001**
**Multivariate analysis with two confounders**				
**Covariate**	**Confounders**	**HR**	**95% CI**	**P-value**
DM	Sex and age	2.468	1.949-3.125	**<0.001**
**Multivariate analysis with all confounders**				
**Covariate**	**Confounders**	**HR**	**95% CI**	**P-value**
DM	Sex, age, BMI, manual work, statin use, alcohol consumption and smoking habits	1.995	1.511-2.633	**<0.001**

Male sex was defined as the reference, both in the univariate analysis and when added as a confounder. In all multivariate analysis, the comparison is individuals without baseline DM.

DM, Diabetes mellitus; TF, Trigger finger; BMI, body mass index; HR, Hazard ratio; CI, confidence interval. P-value < 0.05 was considered statistically significant.

Bold value means statistically significant.

## Discussion

The present observational, longitudinal study showed that DM is an important risk factor for developing TF. When sex, age, BMI, manual work, statin use, smoking and alcohol consumption were added as confounders in the multivariate model, DM remained the main risk factor for TF. Our findings add to the previous knowledge where DM has been associated with a higher incidence of TF (5, 11, 13). This further strengthens the need for including recurring, systematic hand examinations in modern diabetes care.

DM is associated with several mechanisms which could explain the increased risk of developing TF. The flexor tendon and A1 pulley can both be affected by diabetes complications. Formation of advanced glycation end products (AGEs) is the result of hyperglycaemia induced non-enzymatic reaction between glucose and proteins (24, 25). AGEs create pathological collagen cross-links, and are accumulated in tissue with slow turn over, such as tendons (25). The result is a thicker, stiffer, and tougher tendon (23–26).

Furthermore, dysregulation of inflammatory mediators in tendinopathy has been shown in T2D, together with increased apoptosis and formation of fibrous tissue (27). Increased levels of growth factors, disturbed signalling pathways and disrupted interactions in extracellular matrix (ECM) are involved in both types of DM, which might contribute to the development of several hand disorders in the diabetic hand, including TF (26–29).

Individuals with T1D are typically younger at onset than individuals with T2D, and hand complications in individuals with T1D decrease with better glycaemic control (30, 31). A potential long period of insulin resistance, hyperinsulinemia, and dyslipidaemia before a T2D diagnosis, might also contribute to the development of hand disorders (32, 33). It is difficult to assess any differences between T1D and T2D in hand disorders, as many studies lack this information, which is a limitation also for this study. However, studies including only people with T1D or T2D, or reporting their results clearly separated for types of DM, have shown that duration is associated with development of hand disorders in both types of diabetes (34–38).

Besides DM as a risk factor for TF, we also investigated several other known and potential TF risk factors, including age, female sex, BMI, manual work, statin use, and any inflammatory response caused by smoking and/or alcohol consumption. The purpose was to find any other strong associations which should be considered in the multivariate analysis in order to define the impact of DM as a risk factor. We found an increased risk for TF associated with female sex and increased age. Oestrogen and progesterone have previously been shown to be involved in tendon metabolism and healing (32). In this study, with 60% female participants in the MDCS, sex was thus an important confounder when assessing the impact of DM as a risk factor. Age is a known risk factor for TF and could partly be explained with the accumulation of AGEs as a result of normal aging (39). We also observed a small increased HR with BMI as the only covariate. As people with prevalent T2D at baseline show a higher mean BMI, the increased risk could at least partly be explained by the increased risk from DM. However, there could also be a link between higher BMI with potential insulin resistance, hyperlipidaemia, and tendinopathy (40, 41). The role of manual work in the development of TF is controversial, as evidence of job exposure might lead to compensation claims. While biomechanical stressors are associated with TF, age, gender, and medical history are also risk factors (42). Tendinopathy is a known side effect from statin use and was therefore considered a confounder in this study (22, 23). We did not find any correlation with manual work or statin use in this study.

As shown in the present study, there is evidence that DM could be a considerable risk factor for TF. We have chosen to use the term risk factor instead of risk marker, even though we cannot provide true causal evidence according to Bradford Hill’s criteria (43). As described above, previous studies have suggested several ways in which DM affects the tendon and ECM, indicating that there could be a causal relationship. We have also adjusted for other potential causal confounders, which further motivate using the term risk factor.

DM is also a risk factor for development of several other hand disorders that are included in the diabetic hand. Recent studies, including unpublished material, have shown that DM is a risk factor for CTS, UNE, and DC, using the same cohort data as this study (44). However, the cause for this increased risk in people with DM has yet to been proven, where hand disorder prevalence, disease development and severity are most heterogeneous. Further research is needed to determine and explain a causal link between DM and hand disorders, preferably including data regarding type of DM, duration, and glycaemic control.

### Strengths and Limitations

This study has limitations, where lack of information regarding type of DM and DM duration may be the most important ones. Data was collected in the MDCS in the beginning of the 1990s, often described DM as insulin-dependent or non-insulin-dependent. This was practice then and can be seen in other studies as well. In this study, if the participant had a DM diagnosis when recruited to the MDCS, this was defined as baseline DM and duration was not known. Associations with glycaemic control has been shown in other studies (12, 34, 36); unfortunately, this information was not available for all participants included in this study.

Potential bias in this study include detection, selection and reporting bias. Participants in the MDCS with baseline DM would be included in diabetes treatment programs, which might result in TF detection bias, resulting in a higher detection rate for TF as the participants with DM regularly see health care professionals. On the other hand, there was no selection bias when recruiting participants to MDCS cohort, as hand diagnosis were not included in the original scope of the MDCS. Selection bias in the MDCS cohort have been previously described, where participants had a lower mortality compared with controls (45). It is unlikely that this would affect the TF ratio in comparison with the control group. Reporting bias can probably be neglected as TF diagnosis were reported to the NPR without any correlation to the MDCS.

Regarding the statistical method, it is unclear how the large number of censored participants in the Cox PH regression model affects the results. In the statistical model, confounders were chosen based on previous knowledge regarding risk factors for TF together with data availability in the MDCS. Consequently, confounders might be missing due to lack of knowledge and/or data availability.

This study also has several strengths. The MDCS included data from more than 30,000 participants. Participants ages and the follow-up period were relevant for TF, where prevalence increases with age (4). Relevant confounders, such as age, sex, BMI, manual work, statin use, smoking habits, alcohol consumption, were included in the Cox PH regression model.

### Conclusions

In conclusion, the present study showed that DM is an important risk factor for developing TF. When sex, age, BMI, manual work, statin use, smoking and alcohol consumption were added as confounders, DM remained the main risk factor for TF.

## Data Availability Statement

Public access to the data is restricted by the Swedish authorities (Public access to Information and Secrecy Act (https://www.government.se/information-material/2009/09/public-access-to-information-and-secrecy-act/)). Data used in this study can be made available for researchers after special review that includes approval of the research project by both an Ethics committee and the authorities’ data safety committees. Requests to access the datasets should be directed to (https://www.malmo-kohorter.lu.se/malmo-cohorts).

## Ethics Statement

The studies involving human participants were reviewed and approved by Regional Ethical Review Board in Lund, Sweden (DNR: LU51-90; 2009-633; 2019-01439). The patients/participants provided their written informed consent to participate in this study.

## Author Contributions

All authors contributed to the study design. Data analysis was performed by JPL as well as manuscript drafts. All authors agree to be accountable for the content of the work. All authors contributed to the article and approved the submitted version.

## Funding

Regarding the Malmö Diet and Cancer study, this was funded by grants from the Swedish Cancer Society, the Swedish Medical Research Council, AFA insurance, the Albert Påhlsson and Gunnar Nilsson Foundations and the Malmö city council. The funder was not involved in the study design, collection, analysis, interpretation of data, the writing of this article or the decision to submit it for publication. The present study was funded by Skåne University Hospital and local founds at Lund University,the Swedish Diabetes Foundations, the Swedish Research Council (grant number 2021-01942), the Regional Agreement on Medical Training and Clinical Research (ALF) between Region Skåne and Lund University and finally the Stig and Ragna Gorthons foundation.

## Conflict of Interest

The authors declare that the research was conducted in the absence of any commercial or financial relationships that could be construed as a potential conflict of interest.

## Publisher’s Note

All claims expressed in this article are solely those of the authors and do not necessarily represent those of their affiliated organizations, or those of the publisher, the editors and the reviewers. Any product that may be evaluated in this article, or claim that may be made by its manufacturer, is not guaranteed or endorsed by the publisher.

## References

[1] World Health O Global Report on Diabetes. Geneva: World Health Organization (2016). p. 2016.

[2] Banon S Isenberg DA. Rheumatological Manifestations Occurring in Patients With Diabetes Mellitus. Scand J Rheumatol (2013) 42(1):1–10. doi: 10.3109/03009742.2012.713983 23130978

[3] Gutefeldt K Hedman CA Thyberg ISM Bachrach-Lindstrom M Arnqvist HJ Spangeus A. Upper Extremity Impairments in Type 1 Diabetes With Long Duration; Common Problems With Great Impact on Daily Life. Disabil Rehabil (2019) 41(6):633–40. doi: 10.1080/09638288.2017.1397202 29105514

[4] Shah A Rettig M. Trigger Finger Location and Association of Comorbidities. Bull Hosp Jt Dis (2017) 75(3):198–200. 28902605

[5] Koh S Nakamura S Hattori T Hirata H. Trigger Digits in Diabetes: Their Incidence and Characteristics. J Handb Surg Eur Vol (2010) 35(4):302–5. doi: 10.1177/1753193409341103 19687073

[6] Blyth MJ Ross DJ. Diabetes and Trigger Finger. J Handb Surg Br (1996) 21(2):244–5. doi: 10.1016/S0266-7681(96)80106-9 8732409

[7] Makkouk AH Oetgen ME Swigart CR Dodds SD. Trigger Finger: Etiology, Evaluation, and Treatment. Curr Rev Musculoskelet Med (2008) 1(2):92–6. doi: 10.1007/s12178-007-9012-1 PMC2684207 19468879

[8] Brozovich N Agrawal D Reddy G. A Critical Appraisal of Adult Trigger Finger: Pathophysiology, Treatment, and Future Outlook. Plast Reconstr Surg Glob Open (2019) 7(8):e2360. doi: 10.1097/GOX.0000000000002360 31592381 PMC6756654

[9] Vasiliadis AV Itsiopoulos I. Trigger Finger: An Atraumatic Medical Phenomenon. J Handb Surg Asian Pac Vol (2017) 22(2):188–93. doi: 10.1142/S021881041750023X 28506168

[10] Shen PC Chang PC Jou IM Chen CH Lee FH Hsieh JL. Hand Tendinopathy Risk Factors in Taiwan: A Population-Based Cohort Study. Med (Baltimore) (2019) 98(1):e13795. doi: 10.1097/MD.0000000000013795 PMC6344158 30608391

[11] Cagliero E Apruzzese W Perlmutter GS Nathan DM. Musculoskeletal Disorders of the Hand and Shoulder in Patients With Diabetes Mellitus. Am J Med (2002) 112(6):487–90. doi: 10.1016/S0002-9343(02)01045-8 11959060

[12] Vance MC Tucker JJ Harness NG. The Association of Hemoglobin A1c With the Prevalence of Stenosing Flexor Tenosynovitis. J Handb Surg Am (2012) 37(9):1765–9. doi: 10.1016/j.jhsa.2012.06.007 22854253

[13] Chammas M Bousquet P Renard E Poirier JL Jaffiol C Allieu Y. Dupuytren's Disease, Carpal Tunnel Syndrome, Trigger Finger, and Diabetes Mellitus. J Handb Surg Am (1995) 20(1):109–14. doi: 10.1016/S0363-5023(05)80068-1 7722249

[14] Berglund G Elmstahl S Janzon L Larsson SA. The Malmo Diet and Cancer Study. Design Feasibility J Intern Med (1993) 233(1):45–51. doi: 10.1111/j.1365-2796.1993.tb00647.x 8429286

[15] Manjer J Elmstahl S Janzon L Berglund G. Invitation to a Population-Based Cohort Study: Differences Between Subjects Recruited Using Various Strategies. Scand J Public Health (2002) 30(2):103–12. doi: 10.1177/14034948020300020401 12028859

[16] Enhörning S Wang TJ Nilsson PM Almgren P Hedblad B Berglund G. Plasma Copeptin and the Risk of Diabetes Mellitus. Circulation (2010) 121(19):2102–8. doi: 10.1161/CIRCULATIONAHA.109.909663 PMC3763235 20439785

[17] SCB S. Population and Housing Census (1985). Available at: http://share.scb.se/ov9993/data/historisk%20statistik//SOS%201911-/Folk-%20och%20bostadsr%C3%A4kningarna/Folk-%20och%20bostadsr%C3%A4kningen%201965-1990/Folk%20och%20bostadsr%C3%A4kningen%201985%20(SOS)/Folk-o-bostadsrakningen-1985_7.pdf (Accessed March 16, 2021).

[18] Clark TG Bradburn MJ Love SB Altman DG. Survival Analysis Part I: Basic Concepts and First Analyses. Br J Cancer (2003) 89(2):232–8. doi: 10.1038/sj.bjc.6601118 PMC2394262 12865907

[19] Bradburn MJ Clark TG Love SB Altman DG. Survival Analysis Part II: Multivariate Data Analysis–an Introduction to Concepts and Methods. Br J Cancer (2003) 89(3):431–6. doi: 10.1038/sj.bjc.6601119 PMC2394368 12888808

[20] Collins KH Herzog W MacDonald GZ Reimer RA Rios JL Smith IC. Obesity, Metabolic Syndrome, and Musculoskeletal Disease: Common Inflammatory Pathways Suggest a Central Role for Loss of Muscle Integrity. Front Physiol (2018) 9:112. doi: 10.3389/fphys.2018.00112 29527173 PMC5829464

[21] N JHS L A R GVG da Silveira D B PN Almeida SF. Epidemiology of Trigger Finger: Metabolic Syndrome as a New Perspective of Associated Disease. Handb (N Y) (2019) 16(4):542–5. doi: 10.1177/1558944719867135 PMC8283119 31456430

[22] Eliasson P Dietrich-Zagonel F Lundin AC Aspenberg P Wolk A Michaelsson K. Statin Treatment Increases the Clinical Risk of Tendinopathy Through Matrix Metalloproteinase Release - A Cohort Study Design Combined With an Experimental Study. Sci Rep (2019) 9(1):17958. doi: 10.1038/s41598-019-53238-7 31784541 PMC6884518

[23] Kirchgesner T Larbi A Omoumi P Malghem J Zamali N Manelfe J. Drug-Induced Tendinopathy: From Physiology to Clinical Applications. Joint Bone Spine (2014) 81(6):485–92. doi: 10.1016/j.jbspin.2014.03.022 24962977

[24] Giacco F Brownlee M. Oxidative Stress and Diabetic Complications. Circ Res (2010) 107(9):1058–70. doi: 10.1161/CIRCRESAHA.110.223545 PMC2996922 21030723

[25] DeGroot J. The AGE of the Matrix: Chemistry, Consequence and Cure. Curr Opin Pharmacol (2004) 4(3):301–5. doi: 10.1016/j.coph.2004.01.007 15140424

[26] Oliveira RR Medina de Mattos R Magalhaes Rebelo L Guimaraes Meireles Ferreira F Tovar-Moll F Eurico Nasciutti L. Experimental Diabetes Alters the Morphology and Nano-Structure of the Achilles Tendon. PloS One (2017) 12(1):e0169513. doi: 10.1371/journal.pone.0169513 28095484 PMC5240962

[27] Reddy GK. Cross-Linking in Collagen by Nonenzymatic Glycation Increases the Matrix Stiffness in Rabbit Achilles Tendon. Exp. Diabesity Res. (2004) 5(2):143–53. doi: 10.1080/15438600490277860 PMC2496877 15203885

[28] Lundin AC Eliasson P Aspenberg P. Trigger Finger and Tendinosis. J Handb Surg Eur Vol (2012) 37(3):233–6. doi: 10.1177/1753193411421853 21987275

[29] Lundin AC Aspenberg P Eliasson P. Trigger Finger, Tendinosis, and Intratendinous Gene Expression. Scand J Med Sci Sports (2014) 24(2):363–8. doi: 10.1111/j.1600-0838.2012.01514.x 22882155

[30] Lindsay JR Kennedy L Atkinson AB Bell PM Carson DJ McCance DR. Reduced Prevalence of Limited Joint Mobility in Type 1 Diabetes in a U.K. Clinic Population Over a 20-Year Period. Diabetes Care (2005) 28(3):658–61. doi: 10.2337/diacare.28.3.658 15735204

[31] Monnier VM Bautista O Kenny D Sell DR Fogarty J Dahms W. Skin Collagen Glycation, Glycoxidation, and Crosslinking Are Lower in Subjects With Long-Term Intensive Versus Conventional Therapy of Type 1 Diabetes: Relevance of Glycated Collagen Products Versus HbA1c as Markers of Diabetic Complications. DCCT Skin Collagen Ancillary Study Group. Diabetes Control and Complications Trial. Diabetes (1999) 48(4):870–80. doi: 10.2337/diabetes.48.4.870 PMC2862597 10102706

[32] Oliva F Piccirilli E Berardi AC Frizziero A Tarantino U Maffulli N. Hormones and Tendinopathies: The Current Evidence. Br Med Bull (2016) 117(1):39–58. doi: 10.2337/diabetes.48.4.870 26790696

[33] Eid S Sas KM Abcouwer SF Feldman EL Gardner TW Pennathur S. New Insights Into the Mechanisms of Diabetic Complications: Role of Lipids and Lipid Metabolism. Diabetologia (2019) 62(9):1539–49. doi: 10.1007/s00125-019-4959-1 PMC6679814 31346658

[34] Larkin ME Barnie A Braffett BH Cleary PA Diminick L Harth J. Musculoskeletal Complications in Type 1 Diabetes. Diabetes Care (2014) 37(7):1863–9. doi: 10.2337/dc13-2361 PMC4067398 24722493

[35] Raje YR Cracknell G Davoren PM. Frequency of Hand and Shoulder Symptoms in Patients With Type 1 Diabetes. Diabetes Med (2015) 32(7):968–71. doi: 10.1111/dme.12704 25644754

[36] Mathew AJ Nair JB Pillai SS. Rheumatic-Musculoskeletal Manifestations in Type 2 Diabetes Mellitus Patients in South India. Int J Rheum Dis (2011) 14(1):55–60. doi: 10.1111/j.1756-185X.2010.01587.x 21303482

[37] Ramchurn N Mashamba C Leitch E Arutchelvam V Narayanan K Weaver J. Upper Limb Musculoskeletal Abnormalities and Poor Metabolic Control in Diabetes. Eur J Intern Med (2009) 20(7):718–21. doi: 10.1016/j.ejim.2009.08.001 19818294

[38] Gamstedt A Holm-Glad J Ohlson CG Sundstrom M. Hand Abnormalities Are Strongly Associated With the Duration of Diabetes Mellitus. J Intern Med (1993) 234(2):189–93. doi: 10.1111/j.1365-2796.1993.tb00729.x 8340742

[39] Snedeker JG Gautieri A. The Role of Collagen Crosslinks in Ageing and Diabetes - the Good, the Bad, and the Ugly. Muscles Ligaments Tendons J (2014) 4(3):303–8. doi: 10.11138/mltj/2014.4.3.303 PMC4241420 25489547

[40] Tilley BJ Cook JL Docking SI Gaida JE. Is Higher Serum Cholesterol Associated With Altered Tendon Structure or Tendon Pain? A Systematic Review. Br J Sports Med (2015) 49(23):1504–9. doi: 10.1136/bjsports-2015-095100 PMC4680137 26474596

[41] Studentsova V Mora KM Glasner MF Buckley MR Loiselle AE. Obesity/Type II Diabetes Promotes Function-Limiting Changes in Murine Tendons That Are Not Reversed by Restoring Normal Metabolic Function. Sci Rep (2018) 8(1):9218. doi: 10.1038/s41598-018-27634-4 29907811 PMC6003963

[42] Kapellusch JM Garg A Hegmann KT Thiese MS Malloy EJ. The Strain Index and ACGIH TLV for HAL: Risk of Trigger Digit in the WISTAH Prospective Cohort. Hum Factors (2014) 56(1):98–111. doi: 10.1177/0018720813493115 24669546

[43] Hill AB. The Environment and Disease: Association or Causation? Proc R Soc Med (1965) 58(5):295–300. doi: 10.1177/003591576505800503 14283879 PMC1898525

[44] Rydberg M Zimmerman M Gottsater A Nilsson PM Melander O Dahlin LB. Diabetes Mellitus as a Risk Factor for Compression Neuropathy: A Longitudinal Cohort Study From Southern Sweden. BMJ Open Diabetes Res Care (2020) 8(1):e001298. doi: 10.1136/bmjdrc-2020-001298 PMC7199181 32299900

[45] Manjer J Carlsson S Elmstahl S Gullberg B Janzon L Lindstrom M. The Malmo Diet and Cancer Study: Representativity, Cancer Incidence and Mortality in Participants and Non-Participants. Eur J Cancer Prev (2001) 10(6):489–99. doi: 10.1097/00008469-200112000-00003 11916347

